# Effect of Proximal Blood Flow Arrest During Endovascular Thrombectomy (ProFATE): A Multicenter, Blinded-End Point, Randomized Clinical Trial

**DOI:** 10.1161/STROKEAHA.124.049715

**Published:** 2024-12-19

**Authors:** Permesh Singh Dhillon, Waleed Butt, Anna Podlasek, Pervinder Bhogal, Jeremy Lynch, Thomas C. Booth, Norman McConachie, Robert Lenthall, Sujit Nair, Luqman Malik, Tony Goddard, Vinicius Carraro do Nascimento, Emma Barrett, Ketan Jethwa, Kailash Krishnan, Robert A. Dineen, Timothy J. England, for the ProFATE Investigators

**Affiliations:** 1 Interventional Neuroradiology, Queens Medical Centre (P.S.D., N.M., R.L., S.N., L.M.), Nottingham University Hospitals National Health Service (NHS) Trust, United Kingdom.; 2 Stroke (K.K.), Nottingham University Hospitals National Health Service (NHS) Trust, United Kingdom.; 3 Radiological Sciences, Mental Health and Clinical Neuroscience, School of Medicine (P.S.D., A.P., R.A.D.), University of Nottingham, United Kingdom.; 4 National Institute for Health and Care Research Nottingham Biomedical Research Centre (R.A.D.), University of Nottingham, United Kingdom.; 5 Interventional Neuroradiology, Queen Elizabeth Hospital, University Hospitals Birmingham NHS Trust, United Kingdom (W.B.).; 6 Tayside Innovation Medtech Ecosystem, University of Dundee, United Kingdom (A.P.).; 7 Interventional Neuroradiology, Royal London Hospital, Barts Health NHS Trust, United Kingdom (P.B.).; 8 Interventional Neuroradiology, King’s College Hospital NHS Foundation Trust, London, United Kingdom (J.L., T.C.B.).; 9 School of Biomedical Engineering and Imaging Sciences, King’s College London, United Kingdom (T.C.B.).; 10 Interventional Neuroradiology, Leeds Teaching Hospitals NHS Trust, United Kingdom (T.G.).; 11 Interventional Neuroradiology, Gold Coast University Hospital, Australia (P.S.D., V.C.d.N.).; 12 Department of Research and Innovation (Medical Statistics), Manchester University NHS Foundation Trust, United Kingdom (E.B.).; 13 Centre for Biostatistics, Faculty of Biology Medicine and Health, University of Manchester, United Kingdom (E.B.).; 14 Radiology Department (K.J.), University Hospitals of Derby and Burton NHS Foundation Trust, United Kingdom.; 15 Stroke (T.J.E.), University Hospitals of Derby and Burton NHS Foundation Trust, United Kingdom.; 16 Stroke Trials Unit, Mental Health and Clinical Neuroscience, School of Medicine, University of Nottingham, Derby, United Kingdom (K.K., T.J.E.).

**Keywords:** catheters, cerebral infarction, endovascular procedures, stroke, thrombectomy

## Abstract

**BACKGROUND:**

The effect of temporary blood flow arrest during endovascular thrombectomy for acute ischemic stroke is uncertain due to the lack of evidence from randomized controlled trials. We aimed to investigate whether temporary blood flow arrest during endovascular thrombectomy using a balloon guide catheter improves intracranial vessel recanalization compared with nonflow arrest.

**METHODS:**

The ProFATE trial (Proximal Blood Flow Arrest During Endovascular Thrombectomy) was a multicenter, randomized, participant- and outcome-blinded trial at 4 thrombectomy centers in the United Kingdom. Adults with acute ischemic stroke due to anterior circulation large vessel occlusion were randomly assigned (1:1) by a central, Web-based program with a minimization algorithm to undergo thrombectomy with temporary proximal blood flow arrest or nonflow arrest during each attempt. The primary outcome was the proportion of participants achieving near-complete/complete vessel recanalization (expanded Thrombolysis in Cerebral Infarction score of 2c or 3) at the end of the thrombectomy procedure, adjudicated by a blinded independent imaging core laboratory. Analyses were performed on the intention-to-treat population, adjusted for age, IV thrombolysis, onset-to-randomization time, Alberta Stroke Program Early CT Score, occlusion site, randomization site, and National Institutes of Health Stroke Scale.

**RESULTS:**

Between October 10, 2021, and June 27, 2023, we recruited 134 participants, of whom 131 participants (mean age, 75 years; 62 [47%] women and 69 [53%] men) were included in the final analysis. Sixty-six participants were allocated to the temporary blood flow arrest group and 65 to the nonflow arrest group. The proportion of participants with an expanded Thrombolysis in Cerebral Infarction 2c/3 score at the end of the endovascular procedure was 74.4% (49/66) in the flow arrest group and 70.8% (46/65) in the nonflow arrest group (adjusted odds ratio, 1.07 [95% CI, 0.45–2.55]; *P*=0.88). Among the prespecified secondary efficacy outcomes, a lower rate of emboli to a new vascular territory occurred in the blood flow arrest group compared with the nonflow arrest group (1.5% versus 12.3%; adjusted odds ratio, =0.04 [95% CI, 0.01–0.53]; *P*=0.014) and a higher rate of complete recanalization (expanded Thrombolysis in Cerebral Infarction score, 3) after the first attempt in the flow arrest group versus the nonflow arrest group (33.0% versus 15.3%; adjusted odds ratio, =3.80 [95% CI, 1.40–10.01]; *P*=0.007). No between-group differences were identified for the remaining procedural or clinical efficacy (modified Rankin Scale at 90 days) or safety outcomes (worsening of the stroke severity at 24 hours, adverse events, symptomatic intracranial hemorrhage, or mortality).

**CONCLUSIONS:**

Among patients presenting with anterior circulation large vessel occlusion acute ischemic stroke, temporary proximal blood flow arrest during endovascular thrombectomy, compared with nonflow arrest, did not significantly improve the near-complete/complete vessel recanalization (expanded Thrombolysis in Cerebral Infarction score, 2c–3) at the end of the procedure. Larger randomized controlled trials are warranted to confirm or refute a clinically significant treatment effect of temporary flow arrest on the functional outcome following endovascular thrombectomy.

**REGISTRATION:**

URL: https://www.clinicaltrials.gov; Unique identifier: NCT05020795.

Endovascular thrombectomy (EVT) is the standard of care for intracranial large vessel occlusion in acute ischemic stroke (AIS).^1–3^ However, the rate of vessel recanalization remains suboptimal, with only two-thirds of patients achieving near-complete or complete vessel recanalization, a strong predictor of functional outcome.^4,5^ There is ongoing debate over the optimal endovascular reperfusion strategy with European and American recommendations calling for clinical trials to determine the best strategy to achieve vessel recanalization.^2,3^

The use of balloon guide catheters as an adjunctive device during EVT allows intravascular inflation of a compliant balloon, resulting in transient proximal blood flow arrest and even flow reversal when concomitant aspiration is applied in the anterior circulation. This is thought to limit distal clot migration or embolization to new vascular territories during thrombectomy and improve vessel recanalization,^6–8^ which is of clinical relevance as poorer functional outcomes have been observed among patients with distal emboli induced during EVT.^8,9^

Nonrandomized studies have suggested that the use of balloon guide catheters may improve rates of vessel recanalization, first-pass effect (complete vessel recanalization after the first thrombectomy attempt), procedural time, and functional outcomes.^10,11^ However, more recent observational studies have demonstrated similar procedural and clinical outcomes with or without balloon guide catheter use.^12^ Although there are weak recommendations in the current guidelines proposing the use of balloon guide catheters,^2,3^ there remains clinical equipoise, with only 1 in 4 interventionists reporting routine use of balloon guide catheters during EVT.^13^

Due to the lack of high-level evidence from randomized controlled trials, practice heterogeneity, and uncertain effect on outcomes with adjunctive balloon guide catheter use during thrombectomy, this dedicated randomized controlled trial aimed to investigate the effect of temporary proximal blood flow arrest using a balloon guide catheter during EVT on the procedural and clinical outcomes of patients with AIS due to anterior circulation large vessel occlusion.

## Methods

### Data Availability and Sharing

The study protocol, statistical analysis plan, and study data (de-identified participant data and data dictionary) will be considered and made available upon formal request and receipt of a signed material transfer agreement. Requests should be directed to the corresponding author.

### Study Design

ProFATE was an investigator-initiated, pragmatic, multicenter, randomized controlled (1:1), parallel-group, and participant- and outcome-blinded trial comparing temporary proximal blood flow arrest (balloon inflation using a balloon guide catheter) versus no proximal flow arrest (no balloon inflation using a balloon guide catheter) in the extracranial internal carotid artery during EVT for AIS due to large vessel occlusion. Participants were recruited at 4 high-volume (≥150 EVTs per year) EVT centers in the United Kingdom.

The trial received approval of the study protocol and consent forms from the Health Research Authority, the Health and Care Research Wales, and the Wales Research Ethics Committee (Ref: 21/WA/0199) on September 3, 2021. Due to the time-critical nature of performing EVT for eligible patients with an AIS, deferred (2-stage) informed consent was approved by the ethics committee.^14^ The trial was conducted according to the principles of the Declaration of Helsinki and Good Clinical Practice and reported according to the Consolidated Standards of Reporting Trials guidelines. A detailed description of the trial protocol (previously published)^14^ and statistical analysis plan are provided in eSAP 2.

### Participants

This study enrolled functionally independent (prestroke modified Rankin Scale [mRS] score of 0–2) adults (≥18 years) presenting with an AIS with a neurological deficit on the National Institutes of Health Stroke Scale (NIHSS) of at least 2 (NIHSS range, 0–42, increasing values denoting severity) and an intracranial internal carotid artery or middle cerebral artery M1 or proximal M2 branch occlusion. EVT was performed if the Alberta Stroke Program Early CT Score (ASPECTS) was at least 5 on noncontrast computed tomography (ASPECTS range, 0–10, with 1 point subtracted for any early ischemic change in each defined region) without any upper limit of the stroke onset-to-treatment time. Key exclusion criteria included severe stenosis (>90%) or tandem occlusion of the ipsilateral extracranial internal carotid artery prior dissection or previously deployed stents in the ipsilateral carotid artery, posterior circulation occlusion, and participants unlikely to be available for clinical follow-up.

### Randomization and Masking

Participants were randomized using the Pocock and Simon minimization method performed on the electronic case report form developed on Research Electronic Data Capture after obtaining baseline imaging and before the endovascular treatment. Randomization was 1:1 to temporary proximal blood flow arrest (balloon inflation; intervention) or no temporary proximal blood flow arrest (no balloon inflation; control), stratified by use of thrombolysis, and minimized based on key prognostic factors: age (≤70 versus >70 years), time since stroke onset (≤6 versus >6 hours), ASPECTS (≤7 versus >7), clot location (internal carotid versus middle cerebral artery), and stroke severity (NIHSS, ≤12 versus >12).

Core laboratory adjudication of the imaging outcomes was performed by 2 independent assessors (T.G. and V.C.N.). The homogenous use of the type of balloon guide catheter in both groups allowed the raters to adjudicate the imaging outcomes in a blinded fashion. All participants underwent this procedure under conscious sedation/general anesthesia and were blinded to the allocation. Treating physicians performed the clinical assessment at 24 hours posttreatment, and trained specialist nurses performed the telephone follow-up interview at 90 days, both blinded to the treatment allocation. Serious adverse events were reviewed by the 3 members of the data and safety monitoring board, blinded to treatment allocation.

### Procedures

In either trial arm, contact aspiration alone (a direct aspiration first pass technique technique)^15^ or any variation of the combined stentretriever and contact aspiration techniques were permitted as the intended first-line technique during EVT. The “balloon guide with large bore distal access catheter with dual aspiration with stent-retriever as standard approach” technique was recommended if the combined technique was selected as the first line.^16^ Balloon inflation in the extracranial internal carotid artery needed to be performed under direct fluoroscopy, with sufficient inflation required to oppose the vessel wall for adequate flow arrest for ≈30 seconds during each thrombectomy attempt.^17^ Each interventionist involved in the trial had to be familiar with and regularly perform EVT using a balloon guide catheter (≥20/y).

The choice of any endovascular device (catheter, stent retriever, or aspiration device) was left to the discretion of the interventionist, provided the devices were CE-marked. During the trial recruitment phase, the only routinely available CE-marked balloon guide catheter for commercial use in the United Kingdom was the 8Fr Flowgate^2^ catheter (Stryker). Dual aspiration using a continuous vacuum pump and a 60 mL syringe for manual aspiration to both the distal access catheter and the balloon guide catheter at the point of clot retrieval was recommended. At least 3 thrombectomy attempts/passes with or without balloon inflation were required (if needed) according to the randomization outcome, beyond which the use of balloon inflation or a change in guide catheter for any further attempts was at the interventionist’s discretion. The decision for the mode of anesthesia (general anesthesia or conscious sedation) and any rescue therapy, including intracranial angioplasty with or without stenting and intra-arterial pharmacological therapy, was at the discretion of the interventionist.

Before a thrombectomy attempt, cervico-cranial digital subtraction angiography was performed to confirm adequate antegrade flow (particularly in cases of moderate proximal carotid stenosis), the occlusion site, and any presence of emboli in a different vascular territory. Blinded adjudication of the vessel recanalization (extended Thrombolysis in Cerebral Infarction [eTICI]) score, the presence of any distal or new vascular territory emboli, and the presence of carotid dissection or vasospasm were performed for each thrombectomy attempt.

All participants were admitted to a dedicated hyperacute stroke unit and treated according to the National Institute for Health and Care Excellence guidelines,^18^ which included 300 mg aspirin on admission if ineligible for intravenous thrombolysis/alteplase (recombinant tissue type-plasminogen activator at 0.9 mg/kg), adequate blood pressure, and blood glucose control.

Noncontrast CT imaging was performed 24 hours after admission to determine any intracranial hemorrhage. Neurological severity was assessed by trained clinicians using the NIHSS at baseline and at 24 hours after admission. The functional outcome was assessed with the mRS at 90 days by trained specialist nurses using a standardized telephone follow-up interview proforma.

### Outcomes

The primary outcome was the proportion of patients achieving near-complete/complete vessel recanalization (eTICI, 2c–3) at the end of the EVT procedure. eTICI scores denote the degree of vessel recanalization of the affected vascular territory, ranging from 0 (no recanalization) to grade 3 (complete recanalization). Near-complete/complete vessel recanalization (procedural efficacy) was chosen as the primary outcome measure, as it is a key surrogate marker strongly associated with increased functional independence and has been reliably used in previous EVT trials.^4,5,19^

Secondary procedural outcome measures were (1) near-complete/complete recanalization (eTICI, 2c–3), (2) complete recanalization (eTICI, 3) after the first pass, (3) number of thrombectomy attempts to achieve near-complete/complete vessel recanalization (eTICI, 2c–3), (4) procedural time (arterial puncture to final angiography), (5) new or distal vascular territory clot embolization, and (6) change in the automated core infarct volume based on the unenhanced CT imaging between baseline and at 24 hours (to be reported in a subsequent publication).

The clinical efficacy outcomes included the (1) distribution of the mRS score at 90 days, with a shift ranging from 0 (no symptoms) to 5 (severe disability) and 6 (death), and (2) the rate of functional independence (mRS score, 0–2) at 90 days.

The safety outcome measures were (1) early neurological deterioration defined as a change in the stroke severity (NIHSS) of ≥4 between baseline and at 24 hours post-EVT, (2) symptomatic intracranial hemorrhage, defined using the European Cooperative Acute Stroke Study II classification as any intracranial hemorrhage with an increase in the NIHSS ≥4 within 24 hours or death,^20^ (3) procedure-related complications (including vessel dissection/vasospasm/perforation, vascular access site hematoma, or pseudoaneurysm), and (4) all-cause death at 90 days.

### Statistical Analysis

Based on prior observational studies and meta-analyses,^10–12,21–24^ the sample size was estimated by assuming a 25% proportional difference in achieving near-complete/complete vessel recanalization (eTICI, 2c–3) in favor of the balloon inflation group (70%) compared with the noninflation group (45%). Assuming no dropout or loss to follow-up, as the primary outcome measure based on vessel recanalization identified at the end of the procedure would not be affected, and a 4% crossover, a sample size of 124 participants (62 per group), based on the χ^2^ test, was estimated to demonstrate a treatment effect of 80% statistical power at a 2-sided alpha significance level of 0.05. If a patient was enrolled in the trial but, due to spontaneous recanalization, no treatment had taken place or commenced, their trial enrollment was terminated, and instead, another eligible patient was enrolled to ensure sufficient numbers of randomized patients had the procedure undertaken.

Study characteristics were summarized using descriptive statistics for patient demographics, clinical characteristics, comorbidities, and time metrics.

The primary outcome analysis of the near-complete/complete vessel recanalization scores (eTICI, 2c–3) between treatment groups was analyzed using a mixed-effects logistic regression model with adjustment for IV thrombolysis and age, time from stroke onset to randomization, ASPECTS, blood clot location, and NIHSS, with the recruiting site as the random effect.

Secondary outcome measure analyses also used the mixed-effects logistic (binary for dichotomized outcomes) or linear regression models as appropriate using the same adjustment variables as the primary outcome, including the mixed-effects ordinal logistic regression for the full-scale mRS distribution. Analyses of adjusted and unadjusted outcome estimates were expressed as an odds ratio (for logistic and ordinal regression) or a coefficient estimate (for linear regression), both with 95% CIs. A 2-tailed *P* value of <0.05 was considered statistically significant. All analyses were performed on the intention-to-treat population, and the robustness of the primary analysis was assessed in the per-protocol population. Multiple regression imputations were performed for any missing secondary outcome mRS data. No preplanned adjustment for multiple testing was made for analyses of the secondary outcomes.

Prespecified subgroup analyses were based on all minimization variables during randomization, commonly used in stroke clinical trials and reflecting its clinical relevance, including IV thrombolysis, age (≤70 versus >70 years), time since stroke to randomization (≤6 versus >6 hours), ASPECTS (≤7 versus >7), blood clot location (ICA versus MCA), and stroke severity (NIHSS; ≤12 versus >12), were performed. Analysis of the primary outcome in these prespecified subgroups did not comprise the primary analysis and did not inform the sample size calculation. The interpretation of any subgroup effects is based on interaction tests and is hypothesis-generating. Analyses were conducted using StataSE 17.1, and figures were created with the R software 4.2.1.

The previously published study protocol and statistical analysis plan are available in eSAP 2.

### Data Availability

The study protocol, statistical analysis plan, and study data (de-identified participant data and data dictionary) will be considered and made available upon formal request and receipt of a signed material transfer agreement. Requests should be directed to the corresponding author.

## Results

**Figure 1.**
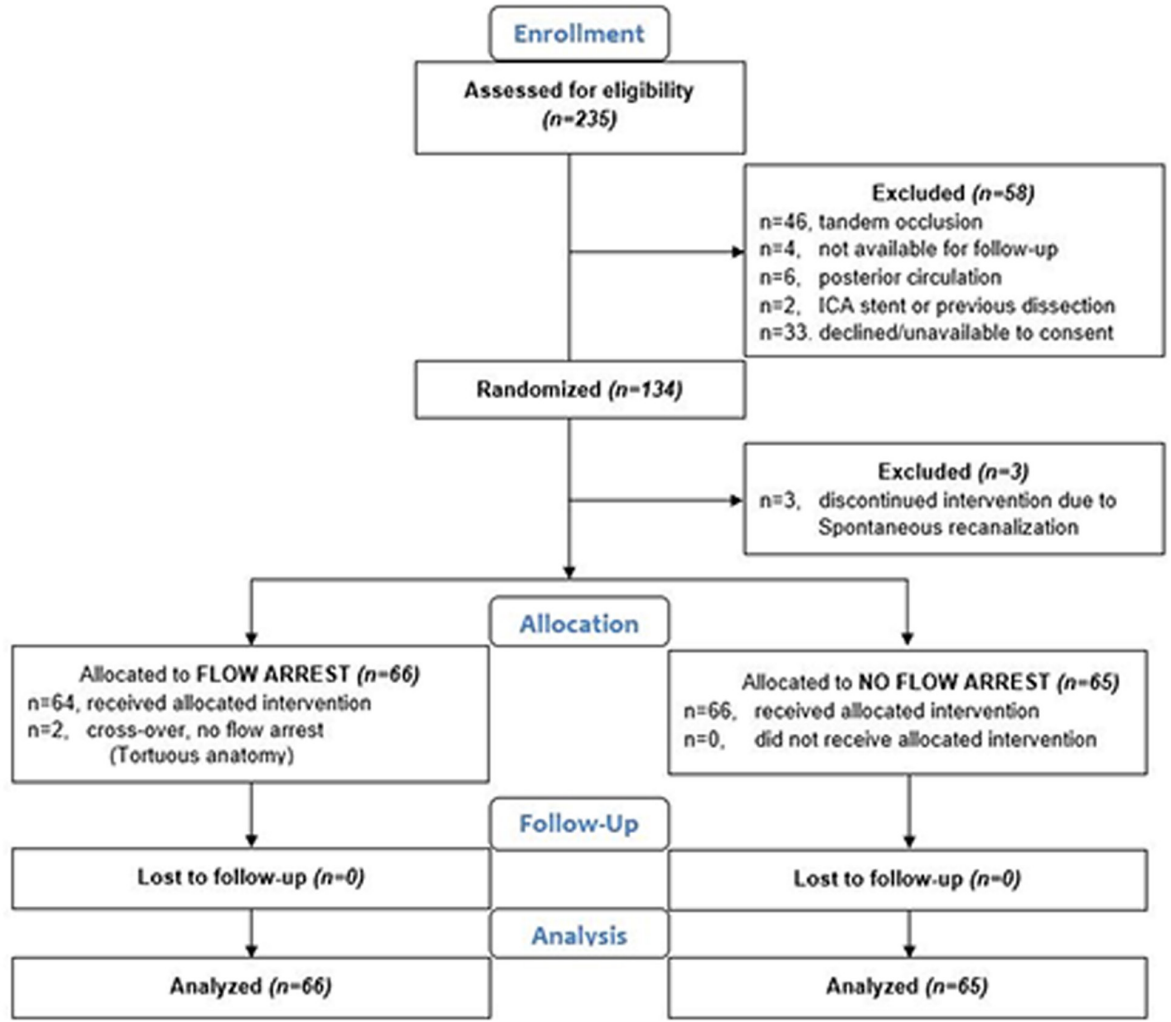
**Flow of participants through the ProFATE trial (Proximal Blood Flow Arrest During Endovascular Thrombectomy).** n indicates number of patients; and Spontaneous recanalization, expanded Thrombolysis in Cerebral Infarction (eTICI, 2c–3).

Between October 10, 2021, and June 27, 2023, a total of 134 participants were randomized in 4 EVT centers. Of these, 3 participants were excluded due to spontaneous vessel recanalization (Figure 1). One hundred thirty-one participants (mean age, 75 years; 62 [47%] women and 69 [53%] men) were included in the primary analysis (66 with blood flow arrest and 65 without). Two participants randomized to the flow arrest group did not undergo temporary flow arrest due to markedly tortuous vascular anatomy necessitating an alternate guide catheter, while all 65 participants in the nonflow arrest group received the intervention as randomized (Figure 1; Table S1). Among the 131 participants included, the median NIHSS score was 16 (interquartile range, 11–20), and the median ASPECTS was 8 (interquartile range, 7–9; Table 1). Median stroke onset-to-randomization time was 391 (interquartile range, 274–809) minutes. A total of 49 participants (37%) received intravenous thrombolysis before EVT. One hundred fourteen participants (87.0%) were treated using the combined technique of stentretriever and contact aspiration. The list of aspiration catheters and stentretrievers used is included in Tables S2 and S3, respectively. Both groups were balanced across the baseline characteristics and comorbidities, except for the proportion of transferred patients to the thrombectomy center and the onset-to-randomization times (Table 1).

**Table 1.**
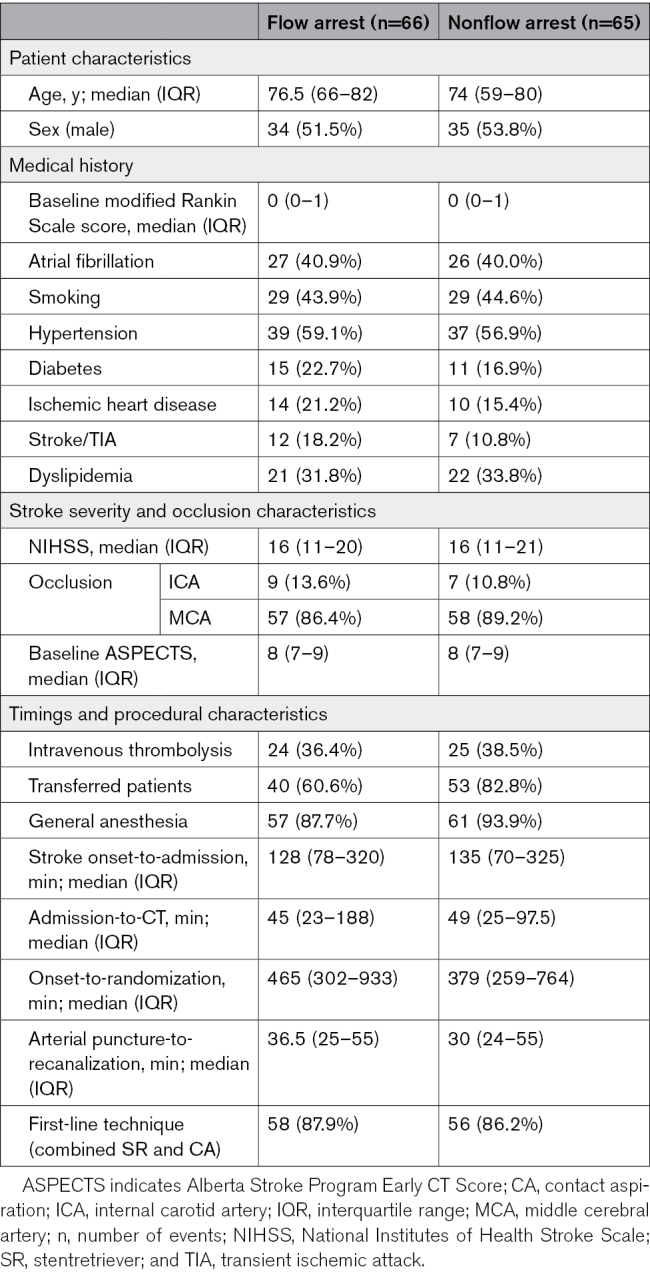
Baseline Characteristics of Participants

The primary efficacy outcome of near-complete/complete vessel recanalization (eTICI, 2c–3) at the end of the thrombectomy treatment was not significantly different between the blood flow arrest and the nonflow arrest groups (74.4% [49/66] versus 70.8% [46/65]; adjusted odds ratio [aOR], 1.07 [95% CI, 0.45–2.55]; *P*=0.88; Table 2; Table S4).

**Table 2.**
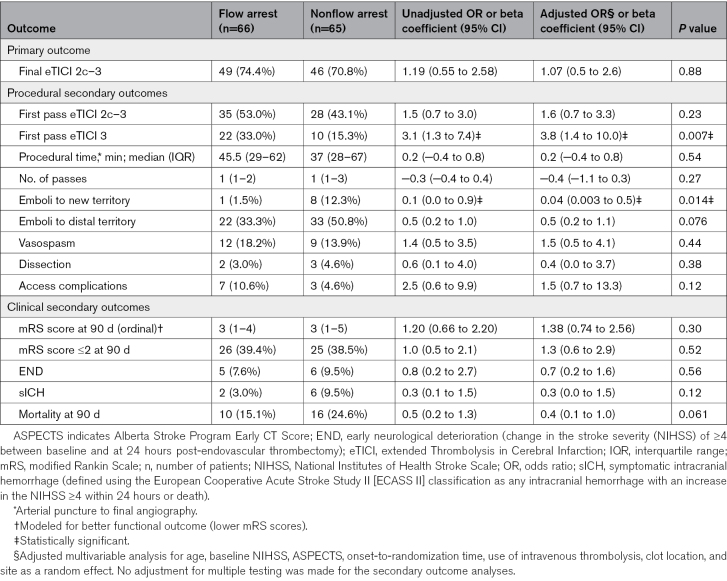
Procedural and Clinical Outcomes

Among the prespecified secondary procedural efficacy outcomes, a lower rate of emboli to a new vascular territory occurred in the blood flow arrest group compared with the nonflow arrest group (1.5% versus 12.3%; aOR, 0.04 [95% CI, 0.01–0.53]; *P*=0.014), and a higher rate of complete recanalization (eTICI 3) was achieved after the first attempt in the flow arrest group versus the nonflow arrest group (33.0% versus 15.3%; aOR, 3.80 [95% CI, 1.40–10.01]; *P*=0.007). No significant difference was observed in the remaining procedural outcomes measures, including the number of thrombectomy attempts or procedural time (Table 2).

**Figure 2.**
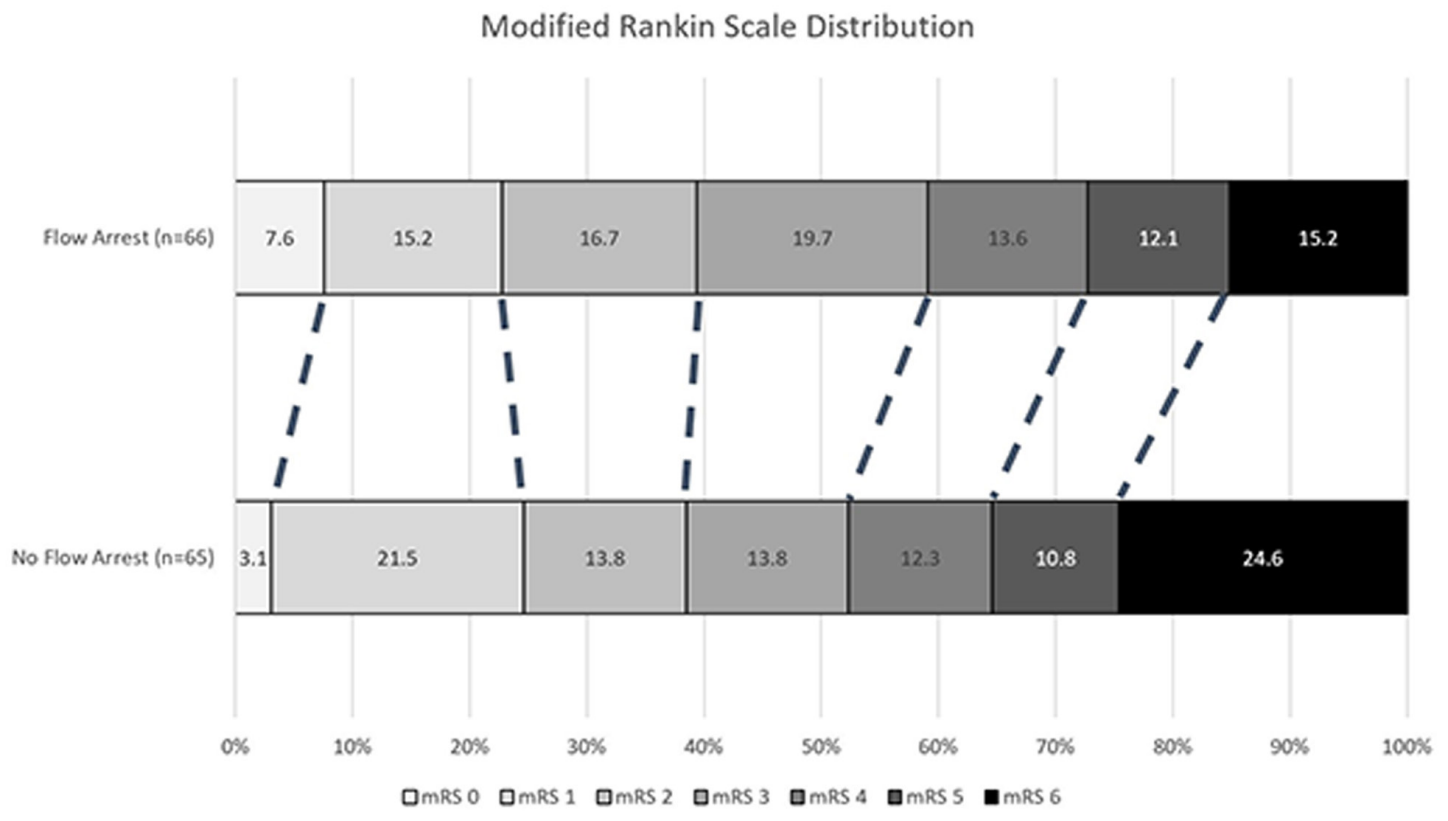
Distribution of the modified Rankin Scale (mRS) at 90 days comparing participants with anterior circulation large vessel occlusion treated with endovascular thrombectomy using temporary blood flow arrest or nonflow arrest.

Temporary blood flow arrest during EVT did not improve the odds of improving the mRS score by at least 1 point at 90 days (common aOR, 1.38 [95% CI, 0.74–2.56]; *P*=0.30) nor increase the odds of achieving functional independence (mRS score of ≤2 at 90 days; 39.4% versus 38.5%; aOR, 1.30 [95% CI, 0.60–2.90]; *P*=0.52; Figure 2; Table 2).

The symptomatic intracranial hemorrhage and mortality rates at 90 days were numerically smaller in the flow arrest group compared with the nonflow arrest group, without reaching statistical significance (symptomatic intracranial hemorrhage: 3.0% versus 9.5%; aOR, 0.30 [95% CI, 0.06–1.50]; *P*=0.12; and mortality: 15.1% versus 24.6%; aOR, 0.40 [95% CI, 0.1–1.01]; *P*=0.06). There were no significant differences in the safety outcomes of early neurological deterioration, procedure-related complications, and adverse events between blood flow arrest and nonflow arrest during EVT (Table 2). The list of adverse events is provided in Table S5.

In the prespecified exploratory subgroup analyses, no heterogeneity in treatment effect on the primary outcome was identified (Figure S1).

## Discussion

In this multicenter randomized trial, temporary proximal blood flow arrest during EVT treatment for participants presenting with anterior circulation large vessel occlusion AIS did not demonstrate improvements in near-complete/complete vessel recanalization (eTICI, 2c–3) at the end of the endovascular procedure, compared with nonflow arrest. However, a lower rate of emboli to a new vascular territory occurred, and a higher rate of complete recanalization after the first attempt was achieved in the blood flow arrest group compared with the nonflow arrest group.

To the best of our knowledge, ProFATE is the first randomized controlled trial to investigate the effect of proximal blood flow arrest during EVT. Near-complete/complete vessel recanalization (procedural efficacy) was chosen as the primary outcome measure as it is a key surrogate marker strongly associated with an increase in functional independence, has been reliably used in previous EVT trials comparing thrombectomy techniques or devices,^4,5,19,25^ and is currently the accepted goal of EVT procedures. The overall rate of eTICI 2c to 3 recanalization demonstrated in the nonflow arrest group (70.8%) was higher than that reported in the HERMES collaboration ([Highly Effective Reperfusion Evaluated in Multiple Endovascular Stroke Trials]; 32%)^1^ and prior trials (45%).^26^ This may be explained by increased operator experience and knowledge from later trials and the predominant use of the combined thrombectomy techniques of stentretriever and contact aspiration (87%) in this trial. These techniques have been associated with higher recanalization rates and are routinely used in modern-day thrombectomy, as opposed to the stentretriever technique only, which was used in most studies included in the prior studies.^1,5,11^

The number of emboli to new vascular territories, a prespecified secondary outcome, was significantly reduced in the proximal flow arrest group compared with nonflow arrest during EVT. This is of clinical relevance as recent studies have demonstrated poorer functional outcomes among patients with distal emboli formed during EVT, which are thought to play a role in microperfusion delay (no-reflow phenomenon) despite successful vessel recanalization.^6,8,9^ Despite the difference in the embolization to new vascular territories rate, no significant improvement in the functional outcome at 90 days was identified, although this trial was not powered to detect these changes. These findings could also potentially be applicable to other neurointerventional procedures where proximal flow arrest may be considered to minimize distal embolic events, for instance during carotid artery stenting for symptomatic carotid stenosis.^27^

Vessel recanalization after the first thrombectomy attempt, the first-pass effect, has also been shown to be an important predictor of functional outcome and is the current objective during the thrombectomy procedure to achieve fast and complete vessel recanalization.^28^ In this study, complete vessel recanalization (eTICI, 3) after the first attempt/pass was higher in the flow arrest group (33%) compared with the nonflow arrest group (15.3%). The mortality rate at 90 days was numerically higher in the nonflow arrest group, which could have, at least in part, been associated with the higher proportion of patients who developed symptomatic intracranial hemorrhage compared with the flow arrest group.

This trial sought to investigate the concept of proximal flow arrest during EVT as opposed to the efficacy of various balloon and conventional guide catheters themselves, which instead was adopted by a separate trial investigating the efficacy of balloon guide catheters.^29^ During the trial recruitment in the United Kingdom, only the 8Fr Flowgate^2^ (Stryker) balloon guide catheter, with an internal diameter of 0.084 inches, was available for commercial use, thereby limiting the compatibility with larger bore aspiration catheters, which have been associated with improved procedural and clinical outcomes. Despite this, the final vessel recanalization rate (eTICI, 2c–3) was in excess of 70% in both groups, higher than that reported in previous studies that included large-bore aspiration catheters.^30^ Furthermore, the mandatory use of the (Flowgate^2^) balloon guide catheter in both the treatment and control arms, the latter performed without balloon inflation, ensured as many variables were standardized as possible to minimize potential confounders by allowing a direct comparison of similarly sized guide and aspiration catheters across both groups.

This study has several limitations. First, the sample size estimate on the procedural efficacy was based on an absolute difference of 25%, and this trial was underpowered to detect smaller between-group differences. However, a smaller difference in the procedural efficacy (eTICI) may not result in a clinically meaningful difference in the functional outcome (mRS at 90 days). For instance, in a recent nonrandomized study,^22^ an absolute difference of 26.5% in the eTICI of 2c to 3 rates (76.8% versus 50.3% between the balloon guide catheter and nonballoon guide catheter groups, respectively) was only associated with a 4% difference in the functional independence rate. Furthermore, such small estimated differences in functional independence rates between groups^12,22^ would necessitate an estimated sample size of over 3000 patients to detect a statistically significant improvement in functional outcome. Second, no preplanned adjustment for multiple testing was made for analyses of the secondary outcomes; hence these findings should be considered exploratory. Although adjustment for multiple covariates for the secondary outcomes was included in the preplanned analysis, only a few events were observed for some of the secondary outcomes. Nevertheless, both adjusted and unadjusted analyses were reported as planned, with the unadjusted analyses demonstrating a similar effect size across the secondary outcomes. Third, the presence of the anterior or posterior communicating arteries may be potential confounders in achieving complete flow arrest for occlusions in the middle cerebral artery. Finally, EVT devices and techniques are continuously evolving, and hence, the findings of this trial, although valid at present, may differ if larger bore guide catheters or newer techniques are utilized in the future.

In summary, among participants presenting with anterior circulation large vessel occlusion AIS, temporary proximal blood flow arrest during EVT, compared with nonflow arrest, did not significantly improve the near-complete/complete vessel recanalization (eTICI, 2c–3) at the end of the endovascular procedure, despite a reduction in emboli to a new vascular territory and a higher rate of complete recanalization after the first attempt. No safety concerns occurred with temporary blood flow arrest in our trial. Larger randomized trials are needed to confirm or refute a clinically significant treatment effect of temporary flow arrest on the functional outcome following EVT for anterior circulation stroke.

## Article Information

### Acknowledgments

The authors would like to thank the data and safety monitoring board and the research collaborators and investigators at the participating sites for their contributions to this trial. They also thank the Neurointerventionalists, Stroke physicians, Mechanical Thrombectomy specialist nurses, and the wider Stroke teams at all participating institutions for the excellent care of all patients. Drs Dhillon, Butt, Dineen, and England were responsible for the conception and design of the study. Drs Dhillon wrote the manuscript. Drs Barrett and Podlasek performed statistical analysis. Dr Dhillon, Dr Butt, Dr Podlasek, Dr Bhogal, Dr Lynch, Dr Booth, Dr McConachie, Dr Lenthall, Dr Nair, Dr Malik, Dr Goddard, Dr Carraro do Nascimento, E. Barrett, Dr Jethwa, Dr Krishnan, Dr Dineen, and Dr England performed the critical revision of the manuscript. All authors approved the final version of the manuscript. ProFATE Research Collaborators: Dr Mahmoud Aboufoul and Dr Gasim Ahmed from the Nottingham site; Dr Saleh Lamin, Dr Han Seng Chew, Dr Benjamin Butler, and Dr Samer Al-Ali from the Birmingham site; Dr Levansri Makalanda, Dr Ken Wong, and Dr Joseph Lansley from the Royal London site.

### Sources of Funding

This investigator-initiated study is independently funded by the Royal College of Radiologists United Kingdom Kodak Scholarship Fund. Dr Dhillon receives funded research time to undertake this trial from the Nottingham University Hospitals National Health Service Trust Research and Innovation Recovery, Resilience and Growth fund (Ref: 21DI004). The funders had no role in the study design, data collection, analysis, interpretation, and writing of the manuscript.

### Disclosures

Dr Dhillon reports grants from Nottingham University Hospitals National Health Service Trust and The Royal College of Radiologists. Dr Bhogal reported travel support from Perflow; stock options in Perfuze and Toro, stock holdings in Ceroflo, compensation from Cerenovus, Balt USA, LLC, Vesalio, Q’apel, Phenox Inc, and Brainomix for consultant services. Dr Booth reported travel support from Balt, consultant services from Medtronic, and core laboratory services from Microvention. The other authors report no conflicts.

### Supplemental Material

Tables S1–S5

Figure S1

Study Protocol

CONSORT Checklist
